# Transient Association Between Elevated NSE and Reduced fT3 Levels Following Mild Traumatic Brain Injury: A 12-Month Prospective Pilot Study

**DOI:** 10.3390/jcm15145676

**Published:** 2026-07-20

**Authors:** Maria Kałas, Mariusz Siemiński, Ewelina Stępniewska

**Affiliations:** Department of Emergency Medicine, Medical University of Gdansk, 80-214 Gdansk, Poland; mariusz.sieminski@gumed.edu.pl (M.S.); e.stepniewska@gumed.edu.pl (E.S.)

**Keywords:** low triiodothyronine, mild traumatic brain injury, neuron-specific enolase, post-concussion symptoms

## Abstract

**Background/Objectives**: In recent years, the perception of mild traumatic brain injury (mTBI) has significantly changed, which implies the need to revise the approach towards post-injury monitoring. Since post-concussion symptoms may overlap with manifestations of neuroendocrine dysfunction, we investigated whether thyroid hormone measurements may provide complementary information to neuron-specific enolase (NSE), a commonly used biomarker of neuronal injury by assessing the association between NSE and thyroid hormone levels following mTBI and to explore potential neuroendocrine alterations during a 12-month follow-up period. **Methods**: We conducted a prospective cohort study. Participants, adults aged ≥18 years with mTBI meeting the inclusion criteria, were recruited from a tertiary trauma center emergency department (ED) in northern Poland from January 2023 to October 2025. During their post-head injury admission to the ED, patients had a blood sample taken and were asked to complete online questionnaires. The procedure was repeated after 3, 6 and 12 months. The NSE, pituitary and thyroid hormones were measured using a chemiluminescence immunoassay method (CLIA). The questionnaires used in the study were: Rivermead Post-Concussion Symptoms Questionnaire (RPQ), Hospital Anxiety and Depression Scale (HADS) and Pittsburgh Sleep Quality Index (PSQI) and the Polish validation of EQ-5D-5L. **Results**: Higher NSE levels were associated with lower fT3 levels at 3 months post injury. No significant association was found at 6- and 12-month follow up. **Conclusions**: In patients with persistently elevated NSE levels three months after mTBI, reduced fT3 concentrations were observed. Although exploratory, these findings suggest a potential link between neuronal injury biomarkers and post-traumatic neuroendocrine responses, supporting further investigation of multi-marker approaches for post-mTBI monitoring.

## 1. Introduction

Traumatic brain injury (TBI) is defined as a brain injury resulting from direct trauma or acceleration–deceleration forces [1]. Its impact on the worldwide health system cannot be underestimated since TBIs are responsible for death and disability more than any other traumatic insult [2]. The annual number of TBI-related visits to emergency departments (EDs) is estimated to be 60 million [3,4]. However, this number does not reflect the full scale of the problem as not every individual suffering from mild TBI seeks medical help [5] and the global incidence of TBI is estimated to be closer to 70 million annually [2]. The leading causes of TBI are motor vehicle traffic, sports and falls, which are currently increasing in numbers with the population aging. The exact proportions vary between high-income countries and low- and middle-income countries; in the latter, road traffic incidents dominate [6].

TBIs are traditionally divided into three categories, mild, moderate and severe, depending on the clinical indicators, such as Glasgow Coma Scale, alteration in consciousness, loss of consciousness (LOC), and post-traumatic amnesia (PTA), related to the trauma [5]. A vast majority, up to 90% of cases, involve mild TBI (mTBI) [1,3,4]. However, mTBI has also been troublesome in terms of post-traumatic care. Despite mTBI being one of the most common neurological diseases, the need for further evaluation of the patients has been neglected for a long time. The nature of the injury was thought to be benign even though recent data suggests that even mild TBI can be associated with impaired life quality and long-term morbidity [7]. Over the past two decades, an increased awareness in the context of follow up and treatment of mTBI has been observed [8]. Nevertheless, in European countries, the rate of post-injury follow up is less than 10% [3]. In their recent study, Eagle et al. proved that 44% of patients with mTBI who did not receive follow-up within 2 weeks post injury had incomplete recovery, 40% had persistent symptoms, and 19% had lower quality of life after one year [9]. After mTBI, 10–20% of the population will experience complications known as post-concussion symptoms (PCSs) [10]. In a 6-month post-mTBI follow up, PCSs were associated with anxiety and post-traumatic stress disorder (PTSD) in 16 to 26.8%. [11,12]. In their study, de Koning et al. [13] proved the importance of post-mTBI follow-up visits regardless of whether the injury required hospitalization or not, as the unfavorable outcome was as high as 20% in non-admitted patients and a quarter of them sought a neurologist consult within 6 months. Thus, the authors postulated that hospital admission should not be a factor in determining aftercare. Moreover, it seems that so far the studies on mild TBI have been poorly representative of the general population and the occurrence of post-concussion symptoms might have been underestimated [7]. A study conducted in a minor head injury clinic confirmed that many patients, even after a mild TBI, experience prolonged PCS, which may result, among other things, in a delayed return to work [14].

As far as post-concussion symptoms are concerned, the complaints reported by patients, such as fatigue, irritability or memory problems, are nonspecific and may also be observed in other medical conditions that arise as consequences of head injury, such as post-traumatic hypopituitarism (PTHP) or other neuroendocrinological disturbances. PTHP, described for the first time in 1918 [15] is most commonly observed in patients who have sustained moderate or severe head trauma, but it may also affect individuals after a mild injury and it seems that its prevalence may be considerably underestimated [15]. The specific PTHP symptoms vary depending on which hormonal axis is disrupted; however, a subset of neuropsychiatric manifestations following TBI like depression, mood disorders, and anxiety have also been linked to PTHP [16]. Moreover, studies involving patients with severe head trauma have suggested that prolonged suppression of the hypothalamic–pituitary–thyroid axis may likewise occur in individuals with less severe injuries [17]. This prompted the hypothesis that the symptoms reported by patients may result either from direct brain injury (which can be assessed by measuring NSE) or from hormone dysfunction secondary to TBI. *Previous studies have reported that abnormalities in free triiodothyronine (fT3) and thyroxine (fT4) levels in patients with traumatic brain injury are associated with poorer neurological outcomes [18]*. *A specific pattern of thyroid hormone dysfunction that may occur following trauma is euthyroid sick syndrome (ESS), which is characterized by reduced serum triiodothyronine (T3) levels in the absence of a compensatory increase in thyroid-stimulating hormone (TSH), and is commonly observed in patients with acute illness [19]. Neuron-specific enolase is a well-established biomarker of neuronal injury, similar to ubiquitin C-terminal hydrolase-L1 (UCH-L1), whereas glial fibrillary acidic protein (GFAP) reflects astrocytic injury and neurofilament light chain (NfL) is a marker of axonal damage. During traumatic brain injury (TBI), acceleration–deceleration forces generate mechanical stress that leads to stretching and disruption of neuronal cell membranes within both neuronal somata and axons. This structural damage results in the early release of intracellular neuronal proteins, including NSE and UCH-L1, into the extracellular space and circulation. In contrast, GFAP and NfL are generally released later, reflecting the subsequent evolution of astroglial and axonal injury* [20,21]. Therefore, the study protocol included parallel monitoring of clinical symptoms along with measurements of NSE and thyroid hormone levels.

The postulated need for post-mTBI follow-up is in accordance with the most recent recommendations which suggest that mTBI should be regarded not as a single event but as a complex and chronic process with potentially long-term consequences [22,23]. Since mTBI evolves over time, the outcome prediction based only on initial radiological findings on clinical presentation may be inaccurate [24]. Indeed, current recommendations require treating mTBI as a complex set of four pillars: a clinical pillar (full GCS and pupillary reactivity); a biomarker pillar (blood-based measures); an imaging pillar (pathoanatomical measures); and a modifier pillar (features influencing clinical presentation and outcome; CBI-M) [22].

At the moment, there are no existing guidelines concerning post-injury management of adults with mTBI first evaluated in emergency departments [9]. Previously, NSE has been investigated for its potential as a prognostic marker in patients with mTBI but nowadays its importance in the diagnosis of the acute phase of mTBI is declining in favor of other biomarkers. Concurrently, research is providing new insight into the etiology of chronic post-mTBI symptoms, which leads to the search for monitoring tools that go beyond the traditional markers of brain damage. Development of post-concussion syndrome is multifactorial and its symptoms such as fatigue, impaired concentration or memory, and depression [25] resemble those found in hypothyroidism. Free triiodothyronine (fT3),which is an active form of triiodothyronine (T3), has an established role in neurogenesis in all stages of brain development [26]. In previous studies, low fT3 levels were correlated positively with mortality in TBI patients [27]. Moreover, the influence of post-traumatic stress disorder (PTSD) on interrupted thyroid function has recently been investigated [28]. Considering this, we decided to study the potential co-existence of PCS and post-traumatic disruptions in the pituitary–thyroid axis and the prospective role of thyroid hormone versus NSE assessments as a monitoring strategy in patients post-mTBI.

## 2. Methods

**Study Setting**: This was a prospective single-center study performed in an emergency department. The ED is localized in a university hospital of 1100 beds, admitting 160,000 patients per year with approximately 38,000 admissions to the ED annually. The ED is one of four EDs functioning in a Tri-City metropolis inhabited by 1,100,000 citizens.

**Patients**: Patients fulfilling the following inclusion criteria were admitted to the study: age ≥ 18 years, meeting the American Congress of Rehabilitation Medicine’s (ACRM) definition of mTBI [29] and having no acute intracranial findings on initial CT. Exclusion criteria were as follows: pregnancy, abnormal post-injury neuroimaging findings *(intracranial hemorrhage: epidural, subdural, subarachnoid, or intraparenchymal hemorrhage and intracerebral hematoma)*, hospitalization > 48 h due to TBI, age < 18 years, lack of conscious consent. Patients were recruited from January 2023 until October 2025.

**Clinical data**: the following demographic and clinical data were noted for each patient: age, sex, mechanism of injury, loss of consciousness, neurological symptoms, comorbidities.

**Clinical scales**: The following clinical scales were used:

*Rivermead Post-Concussion Symptoms Questionnaire (RPQ)*: This is a questionnaire used to measure the intensity of the 16 most common post-head injury symptoms. It is based on a subjective five-scale rating of 0–4, the reference level being premorbid. Zero indicates the absence of a given symptom, while 4 indicates symptoms of high severity. The higher the total score, the more severe the post-concussive symptoms [30]. *The full questionnaire is available in File S1*.

*EQ-5D-5L*: This is an instrument for the measurement of health-related quality of life (HRQL). The EQ-5D-5L consists of a five-dimensional descriptive system questionnaire (mobility, self-care, usual activities, pain/discomfort, and anxiety/depression) and a visual analogue scale (EQ-VAS). Each dimension has 5 levels: no problems, slight problems, moderate problems, severe problems and extreme problems. The patient is asked to indicate his/her health state by ticking the box next to the most appropriate statement in each of the five dimensions. This decision results in a 1-digit number that expresses the level selected for that dimension. The digits for the five dimensions can be combined into a 5-digit number that describes the patient’s health state. The EQ VAS records the patient’s self-rated health on a vertical visual analogue scale where the endpoints are labelled ‘The best health you can imagine’ and ‘The worst health you can imagine’. The VAS can be used as a quantitative measure of health outcome that reflects the patient’s own judgement. The version used in the study was validated for the Polish population [31].

*Hospital Anxiety and Depression Scale (HADS):* This is an assessment of the psychological status of patients with medical issues. It is made of 2 subscales: anxiety (HADS-A) and depression (HADS-D). Each subscale consists of 7 issues concerning symptoms of anxiety and depression. Responses are rated from 0 to 3, with higher scores indicating higher severity. The ratings of the 14 items are summed to yield a total score (0 to 42), or for each subscale separately (0 to 21) [32].

*Pittsburgh Sleep Quality Index (PSQI):* This is a tool used to evaluate sleep quality. It consists of seven components and each component score of this questionnaire ranges from 0 to 3, with 3 indicating the greatest dysfunction or disturbance. All of the component scores are summarized in order to obtain a total PSQI score, which ranges from 0 to 21. The higher the score, the poorer the sleep quality, with a score greater than 5 suggesting significant sleep difficulties [33]. *The full questionnaire is available in File S2*.

**Laboratory assessment**: During the examination, the following laboratory assessments were carried out in the patients:

- NSE. Reference ranges: NSE < 18.5 µg/L.
- *Hormones: free triiodothyronine (fT3), free thyroxine (fT4), and thyroid-stimulating hormone (TSH). Reference ranges: TSH 0.35–4.94 uU/mL; fT3 1.78–7.07pmol/L; and fT4 9.01–19.05 pmol/L*.

All blood sample analyses were performed in the laboratory of the University Clinical Center. *The NSE, pituitary and thyroid hormones were measured using a chemiluminescence immunoassay method (CLIA)*.

**Figure 1:**
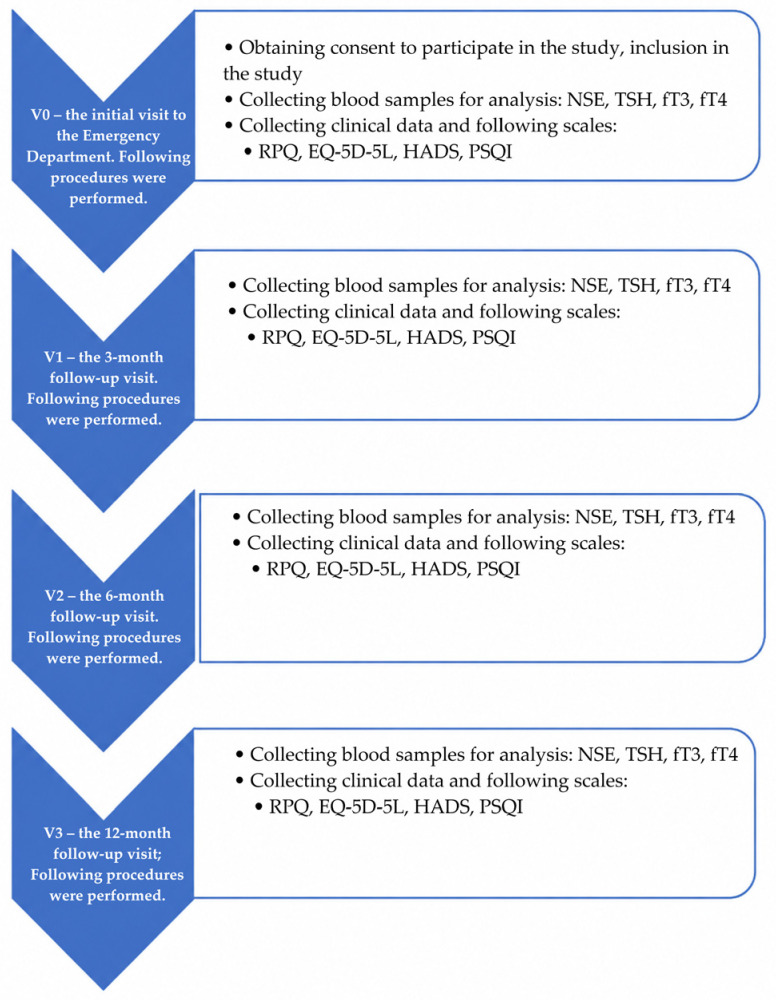
The study protocol.

**Study Protocol**: The following visits were pre-scheduled for each patient (they are presented in Figure 1):

V_0_—the initial visit to the Emergency Department. The following procedures were performed:

- Obtaining consent to participate in the study and inclusion in the study.
- *Collecting blood samples for analysis: NSE, TSH, fT3*, and *fT4*.
- Collecting clinical data and the following scales:
  (a) RPQ;
  (b) EQ 5D-5L;
  (c) HADS;
  (d) PSQI.

V_1_—the 3-month follow-up visit. The following procedures were performed:

- Collecting blood samples for analysis: NSE, TSH, fT3, and fT4.
- Collecting clinical data and the following scales:
  (a) RPQ;
  (b) EQ 5D-5L;
  (c) HADS;
  (d) PSQI.

V_2_—the 6-month follow-up visit. The following procedures were performed:

- Collecting blood samples for analysis: NSE, TSH, fT3, and fT4.
- Collecting clinical data and the following scales:
  (a) RPQ;
  (b) EQ 5D-5L;
  (c) HADS;
  (d) PSQI.

V_3_—the 12-month follow-up visit. The following procedures were performed:

- Collecting blood samples for analysis: NSE, TSH, fT3, and fT4.
- Collecting clinical data and the following scales:
  (a) RPQ;
  (b) EQ 5D-5L;
  (c) HADS;
  (d) PSQI.

### Statistical Analysis

Statistical analyses were performed using non-parametric methods. Comparisons between two independent groups (NSE ≤ 18.5 µg/L vs. NSE > 18.5 µg/L) were conducted using the Mann–Whitney U test. Results are presented as medians and interquartile ranges (IQRs), together with the Hodges–Lehmann estimate of the median difference and Cliff’s delta (δ) effect size. Associations between continuous variables were assessed using Spearman’s rank correlation coefficient (ρ). Changes over time within the same individuals were evaluated using the Wilcoxon signed-rank test, with results reported as median paired differences (follow-up minus baseline). The Shapiro–Wilk test was used to assess the normality of distributions and to justify the use of non-parametric statistical methods. Statistical significance was set at a two-sided α level of 0.05.

The analyses were performed in the Python environment, version 3 (Python Software Foundation, USA), using the following libraries: pandas (pandas-dev, open-source, available at https://pandas.pydata.org), SciPy–the scipy.stats module (The SciPy Community, open-source, https://scipy.org), statsmodels (the statsmodels development team, open-source, https://www.statsmodels.org), and openpyxl (open-source, https://openpyxl.readthedocs.io accesses on 11 October 2025), used for saving and organizing results in Excel spreadsheets. Additional calculations and table preparation were carried out in Microsoft Excel (Microsoft Corporation, Redmond, WA, USA).

## 3. Results

**Figure 2:**
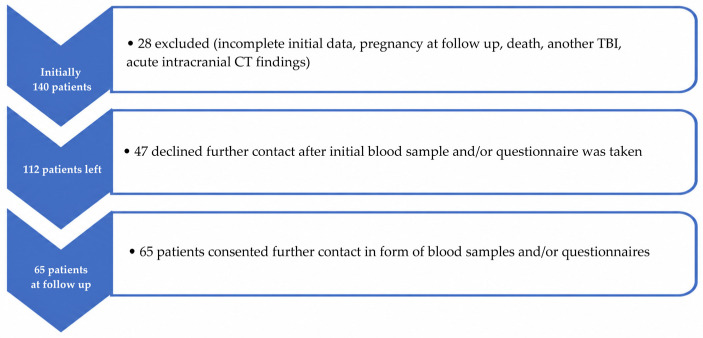
Structure of participants.

Patients were recruited from January 2023 until October 2025. Initially, 140 individuals were enrolled. However, in the course of the study, five patients were excluded due to acute post-injury intracranial findings on CT, one patient was excluded from the control visit due to pregnancy, one patient sustained another severe head trauma, one patient died, and 20 patients had incomplete initial data. Of the remaining 112 people, 65 consented to follow up in the form of blood sample testing and/or questionnaires and 47 patients declined further contact. A total of 19 out of 112 (16.96%) participants declared a past medical history that included thyroid disease. Of the 112 participants ultimately enrolled in the study, 65 presented for laboratory follow-up and/or completed the questionnaires. In this group, 54 participants completed the initial questionnaire. The remaining 47 participants did not attend further follow-up after the initial blood sample was collected. In this group, 23 initial questionnaires were obtained. Patient flow through the protocol-scheduled visits is presented in Figure 2.

Demographic and clinical data of the patients are presented in Table 1.

None of the patients presented with focal neurological deficits.

The most frequent known cause of mTBI was a fall, and the second most frequent cause was a motor vehicle accident (Table 1).

**Figure 3:**
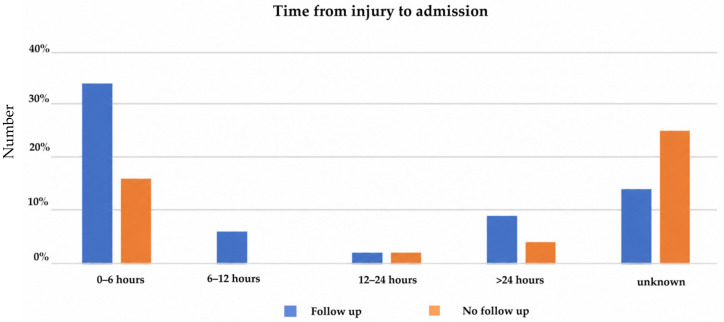
Time from injury to admission.

**Figure 4:**
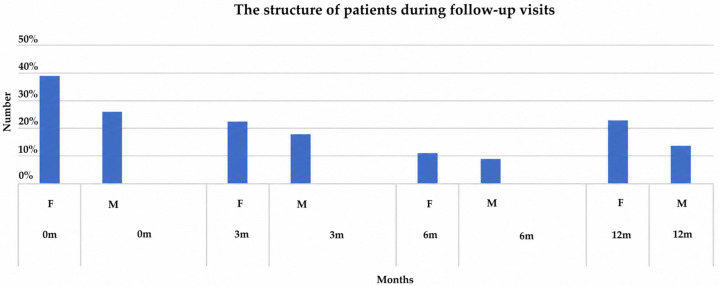
The structure of patients during follow-up visits.

In the group that agreed to participate in the follow-up, the time from injury to presentation was shorter (Figure 3), and at each time point (after 3, 6, and 12 months), the majority were women (Figure 4).

The evolution of the results of clinical scales vs. NSE levels through consecutive visits is presented in Table 2.

Significant changes over time (paired):

(0→3 months): EQ-5D-5L: increase, median = 0.04, *p* = 0.014.

(0→6 months): EQ-5D-5L: increase, median = 0.06, *p* = 0.007. HADS-A: increase, median = 3.00, *p* = 0.034. HADS-D: increase, median = 1.

(0→12 months): EQ-5D-5L: increase, median = 0.06, *p* = 0.001.

**Figure 5:**
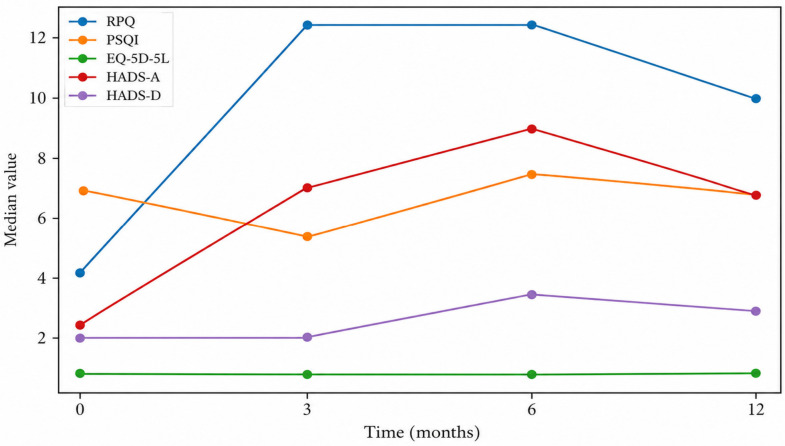
Median value of scales for NSE > 18.5 µg/L.

**Figure 6:**
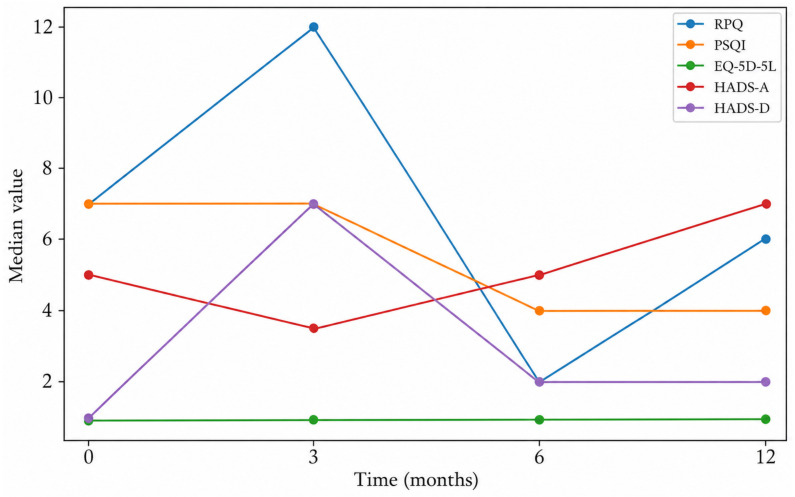
Median value of scales for NSE < 18.5 µg/L.

The above changes applied to patients in both groups, those with NSE >18.5 µg/L (Figure 5) as well as those with NSE <18.5 µg/L (Figure 6).

During the initial post-injury visit, post-concussive symptoms were, as anticipated, more pronounced in patients presenting with elevated NSE concentrations. Notably, during subsequent follow-up assessments, individuals whose NSE levels exceeded the established cutoff demonstrated *lower* Rivermead scores compared with those exhibiting normative values. A comparable pattern was observed for the associations between NSE and HADS-A (at 3 and 6 months) as well as HADS-D (at 6 and 12 months). Furthermore, subjective sleep quality was consistently poorer in later assessments among patients with NSE levels < 18.5 µg/L.

**Figure 7:**
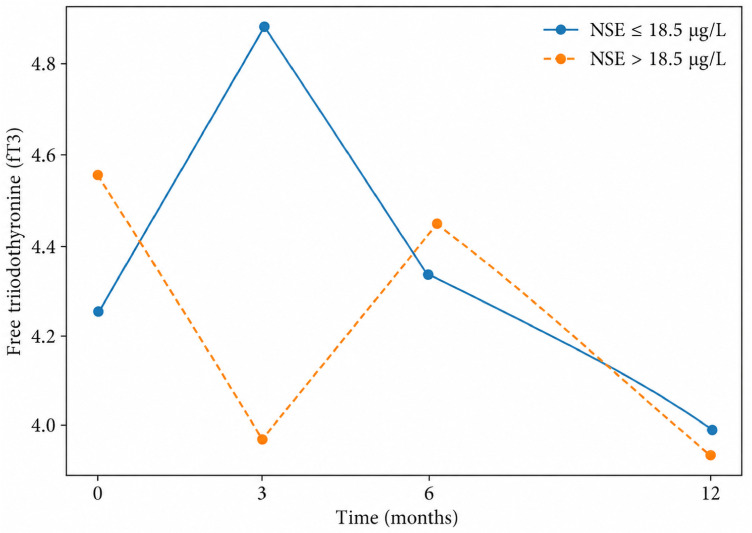
NSE levels vs. ft3 levels over time.

Higher NSE levels were associated with lower fT3 levels at 3 months post injury (Figure 7).

In this study, the interesting phenomenon of NSE changes over time was also analyzed (post 3 months N = 11, post 6 months N = 7, and post 12 months N = 27) (Table 3).

In the cohort of 11 individuals (3 months post injury), stable or increasing NSE values were observed in 63.6% (*n* = 7), whereas a decrease occurred in 36.4% (*n* = 4). Among participants with baseline NSE > 18.5 µg/L, levels remained elevated in 36.4% (*n* = 4), while normalization to ≤18.5 µg/L was observed in 18.2% (*n* = 2). Among those with baseline NSE ≤ 18.5 µg/L, an increase above the threshold occurred in 9.1% (*n* = 1).

In the cohort of seven individuals (6 months post injury), a decrease in NSE predominated, occurring in 85.7% (*n* = 6), while stable or increasing values were noted in 14.3% (*n* = 1). Within the subgroup with baseline NSE > 18.5 µg/L, normalization to ≤18.5 µg/L was more frequent (42.9%, *n* = 3) than persistence of elevated values (14.3%, *n* = 1). An increase above the threshold among those with baseline NSE ≤ 18.5 µg/L occurred in 14.3% (*n* = 1).

In the cohort of 27 individuals (12 months post injury), a decrease in NSE was observed in 70.4% (*n* = 19), compared with 29.6% (*n* = 8) showing stable or increasing levels. Among individuals with baseline NSE > 18.5 µg/L, 22.2% (*n* = 6) maintained elevated values, whereas 40.7% (*n* = 11) normalized to ≤18.5 µg/L. Only 3.7% (*n* = 1) of participants with baseline NSE ≤ 18.5 µg/L exhibited an increase above the threshold.

**Figure 8:**
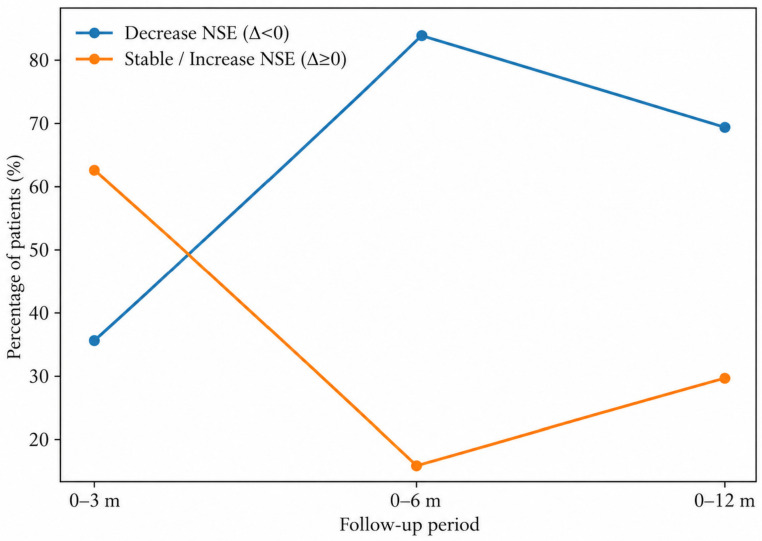
NSE changes over time.

Across all cohorts, decreases in NSE were generally more common than stable or increasing values, except in the 3-month group (N = 11), where stability or increase predominated. In participants with baseline NSE > 18.5 µg/L, normalization occurred more frequently than persistent elevation in two of the three cohorts (N = 7 and N = 27). A small but consistent subset of individuals with baseline NSE ≤ 18.5 µg/L exhibited increases above the diagnostic threshold. In the paired analysis using the Wilcoxon test, it was found that after 12 months (0→12), a statistically significant decrease in NSE levels was observed (Figure 8).

## 4. Discussion

Since mTBIs are a heavy burden on the healthcare system, both in terms of diagnostics and complications, numerous attempts to make it more cost-effective and involve less-radiation exposure for the patients have been made [34]. Biomarkers have been one of the postulated tools that could facilitate mTBI management. According to the FDA (U.S. Food and Drug Administration), the definition of a biomarker is “a defined characteristic that is measured as an indicator of normal biological processes, pathogenic processes, or biological responses to an exposure or intervention, including therapeutic interventions. Biomarkers may include molecular, histologic, radiographic, or physiologic characteristics” [35]. As far as TBI is concerned, we dispose of the indicators of neuronal, glial and axonal cell injury as well as inflammation biomarkers and extracellular vesicles [20]. They have been the subject of various studies assessing their usefulness as decision-making tools for neuroimaging [36] and in terms of long-term prognosis [37,38]. In 2024, two biomarkers, glial fibrillary acid protein (GFAP) and ubiquitin c-terminal hydrolase-L1 (UCH-L1) were approved by the FDA as assisting tools in the decision-making process of post-injury head CT [9].

Enolase is a glycolytic dimeric enzyme that consists of three subunits (α, β, and γ) and five isozymes (αα, ββ γγ, αγ, and βγ) [39]. The isozymes containing a γ subunit are found in neuronal and endocrine tissue and so are named the neuron-specific enolases (NSEs) [40]. Due to the presence in the cytoplasm of neurons [41], NSE has been established as a neuronal cell injury biomarker [20] and it has been considered a potential instrument for post-TBI monitoring. However, current data on its usefulness as a prognostic tool is ambiguous. On the one hand, its value as a prognostic marker in mTBI has been questioned [7]. In our study, the utility of NSE as a marker for monitoring patients following mTBI was likewise not confirmed. Although we observed unexpectedly persistent or even increasing enolase levels over time, we found no evidence that the symptoms reported in questionnaires (RPQ, HADS, and PSQI) by these patients were more pronounced than in the group in which a decline in this biomarker level was observed. Moreover, an increase in self-reported quality of life on the EQ-5D-5L scale was observed, regardless of whether the patient had persistently elevated enolase levels. Neither was the initially promising post-3-month correlation between reduced fT3 and elevated NSE values confirmed in subsequent assessments.

However, an up-to-date meta-analysis by Coelho et al. [42] demonstrated that NSE has predictive value for mortality and functional outcomes in moderate/severe TBI. Moreover, a recent work by Zarei et al. [43] focusing on the diagnostic and prognostic value of NSE and S100B demonstrated that the number of studies including mTBI is inadequate to draw a clinically useful conclusion. What is more, the Zarei et al. [43] study emphasized the fact that there is a lack of studies involving isolated head trauma, whereas extra-cranial injuries are known to influence levels of serum biomarkers. This is consistent with the results obtained in a study by Pelinka et al. which revealed that NSE levels were equally elevated in patients with TBI and multiple trauma without TBI [44]. It may seem, though, that when it comes to assessing the utility of NSE in mTBI, the results negating usefulness in this population might have been premature due to the insufficient number of studies in patients with mTBI. It seems that more studies including larger populations with isolated mild head trauma are needed before drawing a final conclusion.

The discrepancies regarding NSE utility seem, to some extent, to be a result of the detection methods used and the heterodimeric nature of the enolase. As was mentioned above, active enolases are dimers made of non-covalently linked α, β or γ subunits, which are expressed by different genes [45]. Although initially only homodimer γγ was thought to be characteristic of neuronal cells [46], the recent studies proved that heterodimer αγ is also in the NSE form [47,48]. However, αγ can also be commonly found outside the brain [49]. The methods used so far to detect NSE, mainly the enzyme-linked immunosorbent assay (ELISA), were based on antibodies binding to the γ subunit. This means that both γγ and αγ forms of enolase were detected and so the overall result was a combination of γγ and αγ enolase dimers. This may have provoked misleading conclusions regarding neuron-specific enolase levels because simultaneously detected αγ heterodimers are not exclusive to neurons [49]. Indeed, if we analyze the already mentioned systematic review and meta-analysis [43] in terms of detection method, it is noteworthy that in some of the previously conducted studies assessing the potential of NSE as a decision-making or prognostic tool, the method used to detect NSE was the ELISA. This method is less sensitive and specific than the chemiluminescent immunoassay (CLIA) currently used nowadays [50]; however, neither of them can eliminate the risk of false-positive results due to detection of heterodimers. This leads to the conclusion that upcoming studies should be performed using CLIA and in the future, a method specific only to the γγ homodimer would provide more reliable results.

Another factor that seems to have been underestimated in the previous research is the hemolysis index (HI) since it may have a significant impact on the final enolase result. According to Mastroianni et al. [51], even very low hemolysis levels that are not visible (HI 5–30) can increase the value of neuron-specific enolase. It seems reasonable though that every HI should be evaluated for all samples with no evident hemolysis before the NSE assay is done in order to avoid false-positive results. The validity of NSE as a monitoring biomarker following mTBI is further constrained by the potential for falsely elevated concentrations in patients with certain comorbidities, including chronic kidney disease [52]. Finally, because NSE is an established tumor marker, particularly in neuroendocrine neoplasms [53], the detection of elevated levels in asymptomatic patients may lead to costly and unnecessary oncological screening, which was also the case for one of the participants in our study.

Naturally, in the case of our study, the small number of participants does not allow for definitive conclusions, and only a certain trend can be observed. Nevertheless, this illustrates that NSE measurements performed using currently available methods are poor predictors and, consequently, in their present form, do not appear to constitute reliable biomarkers for monitoring patients after mTBI.

Another interesting and statistically significant phenomenon observed in our study was a decrease in fT3 levels at 3 months in the NSE > 18.5 µg/L group. No correlation was detected at 6- and 12-month follow-up. This finding supports the notion that monitoring protocols for patients after head injury should incorporate panels of biomarkers rather than single measurements [37], particularly during the first three months following injury. Although the small sample size precludes definitive conclusions, the observed association may provide a basis for future research exploring the relationship between NSE and thyroid hormone alterations following TBI.

The phenomenon known as euthyroid sick syndrome (ESS), or non-thyroidal illness syndrome (NTIS), first described in the 1970s in critically ill patients, is characterized in this population by low fT3 and/or fT4 levels, elevated rT3 levels, and the absence of an accompanying rise in TSH [54]. It is noteworthy that low T3 is the most characteristic aberration which is present even in the mildest form of NTIS [55]. Initially, the nature of NTIS remained unclear. In recent years, however, advances in molecular techniques, enabling detailed characterization of organ-specific thyroid hormones (THs) transporters, receptors, and deiodinases, have provided evidence that inflammatory signaling pathways play a pivotal initiating role [55,56]. In certain acute conditions, low T3 was correlated with increased mortality [56,57,58]; lately, this finding was identified in patients with COVID-19 [59]. As far as brain injury is concerned, the link between low T3 and illness severity and/or poor outcome was noted for acute ischemic stroke [60,61,62] and severe traumatic brain injury [17]. In a study by Ma et al. [62], the correlation between disrupted thyroid function and an inflammatory process leading to stroke was demonstrated for the first time. Since the role of inflammation in pathogenesis of mTBI is already well-established [37,63] and it was reported that inflammatory markers may remain elevated up to one year post mTBI [64], it permits the supposition that the findings of previous studies could be extrapolated to this population of patients and that T3 may offer potential as a monitoring biomarker following mild traumatic brain injury if confirmed in larger cohort studies. This hypothesis is also supported by previous studies concerning severe traumatic brain injury in which it was suggested that less pronounced and prolonged central down-regulation of the hypothalamic–pituitary–thyroid axis may also be a marker of a less severe TBI [17]. Moreover, recent years have also brought reports of a possible association between post-traumatic stress disorder (PTSD) and thyroid dysfunction, especially in women [28]. PTSD symptoms may partially overlap with PCS [10] and also with complaints related to hypothyroidism. Taking into consideration that thyroid diseases are very common, as demonstrated by our study, in which they were most frequently reported as comorbidities and combining it with the aforementioned data, it seems that the observed association between persistent NSE elevation and lower fT3 levels at 3 months may provide a basis for further studies exploring the role of thyroid hormones in post-mTBI recovery.

The study results also reveal surprising discrepancies regarding the questionnaires used to monitor patient well-being. For four out of the five instruments applied (Rivermead, HADS-A, HADS-D, and PSQI), no statistically significant changes were observed over the course of a year, whereas the EQ-5D-5L score showed a consistent improvement. This raises the question of whether, in the context of patients after mTBI, this latter questionnaire may be too general, or whether, paradoxically, the PCS symptoms experienced by patients do not actually affect their quality of life as much as previously assumed.

## 5. Limitations

The authors are aware that their study has several limitations that warrant its classification as a pilot study. First and foremost, the sample size was relatively small as a result of lower-than-expected attendance at follow-up visits and reluctance to complete questionnaires. *Consequently, the multiple longitudinal comparisons and the relatively small subgroup sizes may have increased the risk of both type I and type II statistical errors*. The second limitation is the fact that the study did not exclude patients with co-occurring injuries which could have influenced NSE levels, and NSE measurements were not corrected for hemolysis levels. Given the characteristics of the emergency department, the ideal timing of obtaining blood samples in terms of thyroid hormones could not always be preserved. In addition, patients exhibiting declining fT3 levels were not subjected to further endocrine assessment or assessment for possible comorbidities, and medications that could affect the thyroid hormone levels were not considered.

## 6. Conclusions

Our study offers an interesting perspective on the use of biomarkers in monitoring patients after mild traumatic brain injury. It highlights the multifactorial nature of mTBI and supports further investigation of biological markers involved in post-traumatic recovery. Although exploratory and *requiring confirmation in larger, multicenter prospective studies with more comprehensive biomarker panels and endocrine assessments*, the observed association between elevated NSE and reduced fT3 levels at 3 months suggests that fT3 may warrant further investigation in future studies as a potential biomarker for monitoring *recovery, facilitating earlier identification of patients at risk of persistent dysfunction, and ultimately improving the long-term care of individuals following mTBI*.

## Figures and Tables

**Table 1:** Baseline demographic and clinical characteristics of the study population (*n* = 112).

Characteristic	Overall (*n* = 112)
Age, years, mean ±SD	46.3 ± 18.2
Female sex, *n* (%)	63 (56.3)
**Comorbidities, *n* (%)**	
Thyroid disease	19 (17.0)
Hypertension	17 (15.2)
Diabetes mellitus	15 (13.4)
Heart disease	12 (10.7)
**Mechanism of injury, *n* (%)**	
Fall	60 (53.6)
Motor vehicle accident	22 (19.6)
Impact with an object	12 (10.7)
Sport-related injury	7 (6.3)
Assault	5 (4.5)
Other	7 (6.3)
**Clinical presentation, *n* (%)**	
Loss of consciousness	35 (31.3)
Nausea and/or vomiting	33 (29.5)
Amnesia	32 (28.6)

Data are presented as mean ± SD or *n* (%).

**Table 2:** Clinical questionnaire scores according to serum NSE concentrations during follow-up.

Scale	Baseline (V0)		3 Months (V1)		6 Months (V2)		12 Months (V3)	
	NSE > 18.5 µg/L	NSE ≤ 18.5 µg/L	NSE > 18.5 µg/L	NSE ≤ 18.5 µg/L	NSE > 18.5 µg/L	NSE ≤ 18.5 µg/L	NSE > 18.5 µg/L	NSE ≤ 18.5 µg/L
RPQ	7	4	12	12.5	2	12.5	6	10
PSQI	7	7	7	5.5	4	7.5	4	7
EQ-5D-5L	0.905	0.931	0.925	0.925	0.941	0.932	0.965	0.974
HADS-A	5	2.5	3.5	7	5	9	7	7
HADS-D	1	2	7	2	2	3.5	2	3

Data are presented as median values.

**Table 3:** Changes in serum NSE concentrations during follow-up.

Follow-Up Interval	Decrease in NSE *n* (%)	Stable/Increased NSE *n* (%)	Persistently >18.5 µg/L *n* (%)	Normalized to ≤18.5 µg/L *n* (%)	Increase Above Threshold *n* (%)
0–3 months (*n* = 11)	4 (36.4)	7 (63.6)	4 (36.4)	2 (18.2)	1 (9.1)
0–6 months (*n* = 7)	6 (85.7)	1 (14.3)	1 (14.3)	3 (42.9)	1 (14.3)
0–12 months (*n* = 27)	19 (70.4)	8 (29.6)	6 (22.2)	11 (40.7)	1 (3.7)

Data are presented as *n* (%). NSE = neuron-specific enolase.

## Data Availability

The data presented in this study are available within the article. Additional information may be obtained from the corresponding author upon reasonable request.

## Supplementary Materials

The following supporting information can be downloaded at https://www.mdpi.com/article/10.3390/jcm15145676/s1, File S1: Rivermead Post-Concussion Symptoms Questionnaire (RPQ); File S2: Pittsburgh Sleep Quality Index Questionnaire (PSQI).

## Abbreviations

CLIA	chemiluminescent immunoassay
ELISA	enzyme-linked immunosorbent assay
ESS	euthyroid sick syndrome
mTBI	mild traumatic brain injury
NSE	neuron-specific enolase
NTIS	non-thyroidal illness syndrome
PCS	post-concussion symptoms
PTSD	post-traumatic stress disorder
fT3	free triiodothyronine
T3	Triiodothyronine
TBI	traumatic brain injury
TH	thyroid hormones
