# Supplementary material for: Transient Association Between Elevated NSE and Reduced fT3 Levels Following Mild Traumatic Brain Injury: A 12-Month Prospective Pilot Study
Source: J Clin Med. 2026 Jul 20;15(14):5676. doi: 10.3390/jcm15145676 (PMC13413295; doi:10.3390/jcm15145676)
Supplement: Supplementary file 1 [file jcm-15-05676-s001.zip › File S1.pdf]

The Rivermead Post-Concussion Symptoms Questionnaire

After a head injury or accident some people experience symptoms which can cause worry or nuisance. We would like to know if you now suffer from any of the symptoms given below. As many of these symptoms occur normally, we would like you to compare yourself now with before the accident. For each one, please circle the number closest to your answer:

0 = Not experienced at all
1 = No more of a problem
2 = A mild problem
3 = A moderate problem
4 = A severe problem

Compared with before the accident, do you now (i.e., over the last 24 hours) suffer from:
1.Headaches 0 1 2 3 4
2.Feelings of dizziness 0 1 2 3 4
3.Nausea and/or Vomiting 0 1 2 3 4
4.Noise sensitivity, easily upset by loud noise 0 1 2 3 4
5.Sleep disturbance 0 1 2 3 4
6.Fatigue, tiring more easily 0 1 2 3 4
7.Being irritable, easily angered 0 1 2 3 4
8.Feeling depressed or tearful 0 1 2 3 4
9.Feeling frustrated or impatient 0 1 2 3 4
10.Forgetfulness, poor memory 0 1 2 3 4
11.Poor concentration 0 1 2 3 4
12.Taking longer to think 0 1 2 3 4
13.Blurred vision 0 1 2 3 4
14.Light sensitivity, easily upset by bright light 0 1 2 3 4
15.Double vision 0 1 2 3 4
16.Restlessness 0 1 2 3 4
Are you experiencing any other difficulties?
1. 0 1 2 3 4
2. 0 1 2 3 4

Reference:

King, N., Crawford, S., Wenden, F., Moss, N., and Wade, D. (1995) J. Neurology 242: 587-592
