# Supplementary material for: Transient Association Between Elevated NSE and Reduced fT3 Levels Following Mild Traumatic Brain Injury: A 12-Month Prospective Pilot Study
Source: J Clin Med. 2026 Jul 20;15(14):5676. doi: 10.3390/jcm15145676 (PMC13413295; doi:10.3390/jcm15145676)
Supplement: Supplementary file 1 [file jcm-15-05676-s001.zip › File S2.pdf]

Pittsburgh Sleep Quality Index

The following questions relate to your usual sleep habits during the past month only. Your answers should indicate the most accurate reply for the majority of days and nights in the past month. Please answer all questions.

1. During the past month, when have you usually gone to bed at night?
2. During the past month, how long (in minutes) has it usually taken you to fall asleep each night?
3. During the past month, what time have you usually gotten up in the morning?
4. During the past month, how many hours of actual sleep did you get at night? (This may be different than the number of hours you spend in bed.)

For each of the remaining questions, check the one best response. Please answer all questions.
5. During the past month, how often have you had trouble sleeping because you:
(A) Cannot get to sleep within 30 minutes
(1) Not during the past month
(2) Less than once a week
(3) Once or twice a week
(4) Three or more times a week
(B) Wake up in the middle of the night or early morning
(1) Not during the past month
(2) Less than once a week
(3) Once or twice a week
(4) Three or more times a week
(C) Have to get up to use the bathroom
(1) Not during the past month
(2) Less than once a week
(3) Once or twice a week
(4) Three or more times a week
(D) Cannot breathe comfortably
(1) Not during the past month
(2) Less than once a week
(3) Once or twice a week

(4) Three or more times a week

(E) Cough or snore loudly

(1) Not during the past month

(2) Less than once a week

(3) Once or twice a week

(4) Three or more times a week

(F) Feel too cold

(1) Not during the past month

(2) Less than once a week

(3) Once or twice a week

(4) Three or more times a week

(G) Feel too hot

(1) Not during the past month

(2) Less than once a week

(3) Once or twice a week

(4) Three or more times a week

(H) Had bad dreams

(1) Not during the past month

(2) Less than once a week

(3) Once or twice a week

(4) Three or more times a week

(I) Have pain

(1) Not during the past month

(2) Less than once a week

(3) Once or twice a week

(4) Three or more times a week _

(J)Other reason(s), please describe. How often during the past month have you had trouble sleeping because of this?

(1) Not during the past month

(2) Less than once a week

(3) Once or twice a week

(4) Three or more times a week

6. During the past month, how often have you taken medicine (prescribed or "over the counter") to help you sleep?

(1) Not during the past month

(2) Less than once a week

(3) Once or twice a week

(4) Three or more times a week

7. During the past month, how often have you had trouble staying awake while driving, eating meals, or engaging in social activity?

(1) Not during the past month

(2) Less than once a week

(3) Once or twice a week

(4) Three or more times a week

8. During the past month, how much of a problem has it been for you to keep up enough enthusiasm to get things done?

(1) No problem at all

(2) Only a very slight problem

(3) Somewhat of a problem

(4) A very big problem

9. During the past month, how would you rate your sleep quality overall?

(1) Very good

(2) Fairly good

(3) Fairly bad

(4) Very bad

Reference:

Buysse,D.J., Reynolds,C.F., Monk,T.H., Berman,S.R., & Kupfer,D.J. (1989). The Pittsburgh Sleep Quality Index (PSQI): A new instrument for psychiatric research and practice. Psychiatry Research, 28(2), 193-213.
